# Genome-wide analysis of the NLR gene family in strawberry reveals a novel immune receptor architecture in Rosaceae

**DOI:** 10.1186/s12864-026-13005-1

**Published:** 2026-06-05

**Authors:** Alicia Sillers, Mishi V. Vachev, Marta Bjornson, Patrick P. Edger, Burkhard Steuernagel, Steven J. Knapp, Mitchell J. Feldmann

**Affiliations:** 1 Department of Plant Sciences, University of California, Davis, One Shields Ave, Davis, 95616 CA USA; 2 Department of Horticulture, Michigan State University, 220 Trowbridge Rd, East Lansing, 48824 MI USA; 3 John Innes Centre, Norwich Research Park, NR4 7UH, Norwich, UK

**Keywords:** NLR, Nucleotide-binding leucine-rich-repeat, Strawberry, Rosaceae, Resistance genes, Effector-triggered immunity

## Abstract

**Background:**

Nucleotide-binding leucine-rich-repeat receptors (NLRs) are key components of plant innate immunity. These intracellular proteins specifically recognize pathogen effectors and subsequently activate a defense response. The genes encoding these proteins comprise one of the largest and most diverse gene families in plants. Understanding the various forms and functions of these proteins is imperative to the goal of generating disease resistant crops. This is particularly important for cultivated strawberry, which is heavily impacted by disease.

**Results:**

Using conserved motifs and functional domains, we identified 827 NLR-encoding genes (NLRs) in the (*Fragaria* *×* *ananassa*) ‘FaRR1’ genome and categorized them according to their association with the anciently diverged RNL, CNL, and TNL classes. Among these, 45 were newly annotated and 149 were predicted to be full-length. 94 genes exhibited a previously unreported architecture, containing both CNL-specific and TNL-specific sequences. This architecture was supported by full-length transcript data and was found to be present in additional Rosaceous species but not in any non-Rosaceous species examined. Expression analyses revealed that 586 NLRs, including nearly all those with the novel mixed architecture, were differentially expressed following inoculation with at least one of five pathogens.

**Conclusions:**

Our genome-wide analysis expands the catalog of NLR genes in cultivated strawberry and uncovers a previously undescribed form of domain organization that may be unique to the Rosaceae family. The results suggest that recombination or domain swapping among N-terminal regions has contributed to NLR diversification in this lineage, providing insight into the evolutionary and functional basis of pathogen recognition in strawberry.

**Supplementary Information:**

The online version contains supplementary material available at 10.1186/s12864-026-13005-1.

## Introduction

Cultivated strawberry (*Fragaria* *×* *ananassa*) is an allo-octoploid perennial crop of global economic and nutritional importance. In the United States, it consistently ranks among the top three non-citrus fruits in terms of production volume and market value [1]. However, strawberry cultivation is constrained by numerous fungal, bacterial, and viral pathogens that substantially limit yield and quality [2]. Understanding the genetic basis of disease resistance and the natural variation in strawberry resistance genes is essential for developing cultivars with durable resistance and ensuring sustainable production.

Plants have evolved a suite of complex mechanisms for protecting themselves against pathogen infection. The first line of defense is mechanical, with structures such as the cuticle and cell wall serving as physical barriers to infection [2]. When pathogens come into contact with the cell membrane, pattern recognition receptors (PRRs) embedded in the membrane can trigger the next line of defense: pattern-triggered immunity (PTI) [3]. In this process, pathogen-associated molecular patterns (PAMPs) are recognized and result in induction of immune response pathways, typically including reinforcement of the plant’s physical barriers [2].

Some pathogens have evolved the ability to counteract this process by secreting effector molecules into plant cells, which function in suppressing PTI [3]. This leads to the next line of defense, effector-triggered immunity (ETI) [2]. The most prevalent genes that encode proteins responsible for initiating this stage of immunity are the nucleotide-binding leucine-rich-repeat receptor-encoding genes, or NLRs [2]. The intracellular NLR proteins recognize secreted effectors and trigger a second immune response. This response typically includes localized cell death, known as the hypersensitive response, as well as induction of proteins and hormones that aid in combating pathogens within the plant [2, 4, 5].

There are three anciently diverged NLR classes that differ in their exon-intron structures and N-terminal domains [6, 7]. These are the TIR-type NLRs (TNLs), which have a Toll/interleukin-1 receptor (TIR) domain at the N terminus; CC-type NLRs (CNLs), which have a coiled-coil (CC) domain at the N terminus; and RPW8-type NLRs (RNLs), which have an N-terminal domain resembling the *RESISTANCE TO POWDERY MILDEW 8* gene [6–9]. The different N-terminal domains each play a role in signaling, allowing activation of downstream pathways once a pathogen has been detected [7].

Following the class-specific N-terminal domain is the nucleotide binding site (NBS) domain, specifically known as the NB-ARC domain due to its NB and ARC subdomains [10]. This domain acts as a molecular on/off switch for the NLR. It has been proposed that, by binding and hydrolyzing ATP, the NB-ARC domain causes a conformational change of the whole protein, affecting its catalytic state [11]. Finally, at the C terminus, there is a leucine-rich repeat (LRR) domain. This is the domain responsible for specific recognition of effector molecules [4].

In addition to these canonical domains, some NLRs have been observed with integrated domains downstream of the LRR region [7, 12]. These domains are thought to be incorporated through sequence exchange in gene clusters and can contribute to diverse mechanisms of pathogen recognition, such as by acting as “baits” for effector molecules [7, 12]. These mechanisms often involve complexes of multiple NLRs, in which one protein, known as a “sensor”, binds the effector, and another, known as a “helper”, signals once binding has occurred [12]. NLRs not involved in binding, or indirect binders, usually have less diversity in their LRR domains, or may lack an LRR domain altogether [13–15]. The activated helpers, either directly or through downstream signaling partners, form a ‘resistosome’ oligomer which can insert in the plasma membrane to cause ion imbalance, cell death, and disease resistance [16].

Although NLRs have been described in many plant genomes, their full diversity and evolutionary history remains poorly understood in strawberry. Here, we conducted a genome-wide analysis of the (*F.* *×* *ananassa*) ‘Royal Royce’ reference genome to identify and classify NLR genes. We identified 827 putative NLRs, including 45 that were previously unannotated, and, notably, discovered a previously unreported domain architecture that has, so far, only been observed in the Rosaceae lineage. This work expands our understanding of NLR evolution in plants and highlights genes that could contribute to durable disease resistance in strawberry.

## Methods

### *Fragaria **×** ananassa* NLR gene prediction with NLR-Annotator

We used the program NLR-Annotator to carry out de novo identification of NLR-associated loci in the strawberry ‘Royal Royce’ reference genome, ‘FaRR1’ [17, 18]. The returned loci were then intersected with the ‘FaRR1’ gene models from annotation version 1.0 using Bedtools2 [19]. We manually inspected loci that did not intersect any gene models in order to determine whether these loci were potentially functional genes or whether they were more likely to be pseudogenes. Unannotated loci were translated into all six reading frames using the Expasy translate tool, and each reading frame was analyzed with Interpro to check for functional domain-encoding open reading frames [20, 21]. When a locus appeared to have one or more premature stop codons, we inferred the intron-exon structure by comparing the sequence of the locus to the coding and non-coding sequences of syntenic genes. Loci that appeared potentially functional were manually added to the gene models, while those that appeared to be pseudogenes were excluded from further analyses.

### RNL prediction

RNLs were separately identified by conducting a Pfam search of the peptide sequences for the whole ‘FaRR1’ proteome using Interpro [21]. This process was annotation-dependent. We filtered the results to include only those containing at least one RPW8 domain. All genes with this domain present were categorized as RNLs.

### Functional domain prediction

The peptide sequences of all putative NLRs identified by the above methods were used as input for an Interpro search that included the Pfam and COILs databases [21–23]. The domains were quantified, and this quantification was used for selection of example genes to represent different common domain architectures. The coordinates of the domains were used for visualization of these representative genes using the program Domain Graph 2.0 [24]. The diagrams were further arranged and edited using BioRender. Additionally, non-canonical domains were quantified and visualized in R using the ’ggplot’ and ‘ggbreak’ packages [25, 26].

### Sorting of genes into NLR class-associated subgroups

We carried out two rounds of sorting to group putative strawberry NLR genes into five subgroups based on association with the three NLR classes. Before intersecting with gene models, NLR-Annotator loci were sorted into CNL, TNL, Mixed architecture (MNL), and Other categories based on presence of CNL-specific and TNL-specific motifs using custom R scripts [27]. Loci from each group were intersected with the gene models separately, such that the gene lists retained the original groupings. Genes in each of these groups were then further sorted based on presence of TIR and CC domains, and final categorizations were determined based on a combination of motif and domain data. RNLs were known to have RPW8 domains as a prerequisite but were further sorted based on their other canonical domains. During the sorting process, we also filtered out genes that intersected NLR-Annotator loci but were not predicted to have any canonical NLR domains.

Based on the combined motif and domain data, genes could be categorized as MNLs based on presence of a TIR domain in combination with either a CC domain or a CNL-type NB-ARC domain. CNL-type NB-ARC domains were identified by the presence an RNBS-D motif and/or an RNBS-A motif, which are labeled as motif 2 and motif 6 in the NLR-Annotator output [17, 28]. As the annotations of loci returned by NLR-Annotator sometimes diverged from ‘FaRR1’ gene annotations, MNL genes predicted to have CNL-type NB-ARC domains and no CC domains were manually inspected in IGV to ensure that the aforementioned motifs fell within the gene annotations [29]. Additionally, genes with TIR and CC domains but no RNBS-D or RNBS-A motifs were manually inspected to ensure that the predicted CC domains resembled true CC N-terminal domains. This was done by checking that the CC domains were upstream of the NB-ARC and did not overlap any other domains.

### NLR gene prediction with BLAST

We carried out additional NLR identification with BLAST in order to check for NLR-encoding regions that may have been missed by NLR-annotator. To do this, canonical NLRs were selected from different subgroups, and the sequences of their NB-ARC domains were used as queries for tblastn searches of the ‘FaRR1’ reference genome. The genes selected were the RNL gene Fxa1Dg201244, the Rx-like CNL gene Fxa7Dg101019, the non-Rx CNL gene Fxa6Dg104044, the TNL gene Fxa3Ag104126, and the MNL gene Fxa3Bg203787. We used an e-value cut-off of 1e-06 and a percent identity cut-off of 60. We investigated BLAST hits that were not in our gene list by inspecting the gene annotations and checking for NLR functional domains with Interpro [21]. Inspection of gene annotations and loci without annotated genes followed the same methods as those described for inspecting NLR-annotator loci that did not overlap any annotated genes.

### Analysis of MNL architecture with Iso-Seq data

RNA from roots of the *F*. *×* *ananassa* cultivar ‘UC Moxie’ was used to generate full-length cDNA data using PacBio Iso-Seq long-read RNA sequencing (PRJNA1365949). The Iso-Seq reads were mapped to the ‘FaRR1’ reference genome using minimap2 and visualized in IGV [29, 30]. Isoforms that were observed in multiple reads of the MNL genes Fxa5Ag200278 and Fxa1Cg100403 were diagrammed using Biorender.

### Phylogenetic tree construction

The NB-ARC sequences of all strawberry NLR genes were extracted using the coordinates from the Interpro output and subsequently aligned with MUSCLE using default settings [31]. The alignment file was then used to create a maximum likelihood phylogenetic tree using RAxML-NG with an LG+G4+F model and autoMRE bootstrapping setting [32]. This tree was rooted at midpoint in the Interactive Tree Of Life (ITOL) web tool, then uploaded to R for analysis of subgenome enrichment within phylogenetic clades [33]. Enrichment was assessed with permutation-based testing, in which gene labels were randomly reassigned across the tips of the phylogenetic tree 10,000 times. For each permutation, the maximum enrichment statistic was recorded for generation of a null distribution, controlling for multiple testing across all nodes. The actual distribution was compared to the null in order to determine the boundaries of clades with statistically significant enrichment of one NLR subgroup. The tree was then visualized in ITOL, with enriched clades annotated as colored ranges. The subgroup of the gene of each NB-ARC domain was indicated with a corresponding branch color. The tree was converted to a cladogram to maximize visibility of these branch colors. Additionally, the NB-ARC sequences of genes identified with NLR-Annotator and Interpro were analyzed with MEME from the MEME suite [34]. MEME was used in classic mode, and the lists of sequences matching each motif were extracted and annotated with subgroup data in order to identify motifs specific to the MNL-enriched Clade.

### Identification and classification of NLRs in eleven additional species

Publicly available genomes, gene annotations, and protein fasta files were downloaded for *Fragaria vesca* [35], *Fragaria iinumae* [36], *Rosa chinensis* [37], *Rubus occidentalis* [38], *Malus domestica* [39], *Prunus dulcis* [40], *Arabidopsis thaliana* [41], *Vitis vinifera* [42], *Solanum lycopersicum* [43], *Oryza sativa* [44], and *Marchantia polymorpha* [45]. In each of these genomes, NLRs were identified and classified as described for *F.* *×* *ananassa*. This included identification of MNLs based on presence of a TIR domain in combination with a CNL-type NB-ARC domain and/or an N-terminal CC domain that does not overlap any other domains. However, MNL gene annotations in these other species were not verified with Iso-Seq data. Phylogenetic relationships among the species were determined based on prior study [46–48]. A diagram including a cladogram and table was created using Biorender. MNLs for each species were checked for the presence of Rx CC domains with Interpro and for the presence of the two MNL-specific motifs using MAST from the MEME Suite [21, 34].

### Identification of NLR gene clusters

Gene clusters are defined here as groups of genes in which every NLR is within 200Kb of another NLR gene, based on methods used in previous studies [28, 49]. In order to identify clustered genes, we used Bedtools2 to intersect the NLR genes with 200Kb sliding windows [19]. Windows with less than two genes were filtered out, and sliding windows were manually evaluated for duplicate and overlapping clusters to generate the final gene cluster list.

### dN/dS analysis

We calculated the rates of synonymous and non-synonymous mutations for syntenic gene pairs across the ‘FaRR1’ genome using SynMap2 from the CoGe comparative genomics platform [50, 51]. This tool was run six times with each unique pairing of the four strawberry subgenomes as input in order to get all possible pairs of homoeologous genes. Output files were then filtered to retain NLRs, cytochrome P450s (CYPs), aconitases (ACOs), and oxoglutarate dehydrogenases (OGDHs). We also calculated the rates of synonymous and non-synonymous mutations for paralogs using the PlantTribes2 pipeline [52]. This process began with BLASTP and HMMER to identify orthogroups, followed by sequence alignment, trimming, and removal of fragmentary sequences. Integrated gene family FASTA files were then used to generate protein and codon-aware multiple sequence alignments. Finally, we employed CodeML from the PAML package to calculate dN and dS values [53]. We carried out statistical analysis of dN/dS values among the different groups of genes using the non-parametric Kruskal-Wallis test with post-hoc Dunn’s test for means separation, and visualized dN/dS distributions with the ‘ggplot2’ R package [25].

### Differential gene expression analysis

The following Illumina RNAseq reads were downloaded from the NCBI Sequence Read Archive: strawberry leaves inoculated with *Colletotrichum fructicola* (PRJNA362483) [54], strawberry leaves inoculated with *Colletotrichum gloeosporioides* (PRJNA754844) [55], strawberry leaves inoculated with *Xanthomonas fragariae* (PRJNA633051 and PRJNA603963) [56, 57], and strawberry flowers inoculated with *Botyrtis cinerea* (PRJNA761556) [58]. Strawberry leaves inoculated with *Podosphaera aphanis* were downloaded from the China National Center for Bioinformation (PRJCA001734) [59]. All reads were trimmed with Trimmomatic [60] using ‘SLIDINGWINDOW:4:20 MINLEN:50’ and aligned to the ‘Royal Royce’ genome ‘FaRR1’ using HISAT2 v2.21 [61] with default parameters. The resulting SAM files were filtered with SAMtools [62] for quality score of at least 40. Read counts for each gene were computed with featureCounts [63] and used as input for DESeq2 [64] for differential gene expression analysis. The default analysis was run as described in the DESeq2 vignette [65] to contrast resistant and susceptible samples at each time point. Log$_2$(fold change) shrinkage for visualization was performed with the “apeglm” method for effect size shrinkage [66]. Visualization was performed with the R package ‘ggplot2’ [25]. An ANOVA test was run on the percentages of genes in each subgroup that were differentially expressed in response to pathogen infection in five different infection datasets. This was followed by a Tukey test to determine which pairwise comparisons of subgroups were significant.

## Results

### Genome-wide NLR prediction

To carry out our initial search for NLRs, we used the program NLR-Annotator, which identifies NLR loci de novo using a set of 20 possible conserved motifs described in Jupe et. al. (2012) [17, 28, 67]. The different motifs are located in varied regions across NLR genes and include general motifs as well as sequences that are specific either to CNLs or TNLs [28]. This process resulted in identification of 919 putative NLR loci across the ‘FaRR1’ strawberry reference genome (Additional File 1: Table S1) [18].

We intersected the loci with the ‘FaRR1’ gene models using Bedtools2 in order to get gene annotation information and improve prediction of gene coordinates [19]. There were 62 loci that did not overlap with any annotated genes. These were manually inspected by translation of the sequences into all six reading frames, comparison of the sequences to syntenic genes to predict intron-exon structure, and prediction of functional domains in open reading frames. Seventeen of the unannotated loci were determined to be probable pseudogenes while the remaining 45 were manually annotated and added to the list of putative NLR genes.

We carried out a separate search for RNLs, as NLRs of this class can be missed during de novo prediction by NLR-Annotator. We used Interpro to predict functional domains across annotated genes of the ‘FaRR1’ genome using the Pfam database and then filtered the results to genes containing the RPW8 domain [21, 22]. Ninety-nine genes, all of which were previously annotated, were predicted to have this domain and were thus characterized as RNLs.

Finally, we conducted tblastn searches using the NB-ARC domains of five canonical NLR genes representing different NLR subgroups (Additional File 2: Table S2). These searches collectively resulted in 35 hits that were not already in our gene list, but only four of these were determined to be NLR-encoding. These four genes were added to our list.

### Analysis of conserved motifs and functional domains

Conserved motif data was obtained from the NLR-Annotator output, and predicted functional domains were obtained from an Interpro search of the NLR peptide sequences using the Pfam and COILS databases (Additional File 3: Table S3) [17, 21–23]. We used this data to categorize NLR genes according to their predicted domain architecture, as well as to filter out genes that appeared to have been incorrectly identified as NLRs. This resulted in a final list of 827 putative NLR genes, 149 of which were predicted to be “full-length” with at least one N-terminal signaling domain, NBS domain, and LRR domain (Table 1, Additional File 4: Table S4).

**Table 1:** Number of full-length and partial NLR genes in each of five domain architecture subgroups

Domain architecture	Number of genes
RPW8	57
RPW8-NBS	42
**Total RNLs**	**99**
CC	25
CC-NBS	178
CC-NBS-LRR	27
**Total CNLs**	**230**
TIR	83
TIR-NBS	153
TIR-NBS-LRR	62
**Total TNLs**	**298**
TIR-CC	5
TIR-CC-NBS_TNL_	1
TIR-CC-NBS_CNL_	9
TIR-NBS_CNL_	19
TIR-CC-NBS_CNL_-LRR	12
TIR-NBS_CNL_-LRR	48
**Total MNLs**	**94**
NBS	89
NBS-LRR	17
**Total Other NLRs**	**106**
**Total NLRs**	**827**

Quantification is based on the presence or absence of canonical NLR domains. Subgroups are assigned based on association with the three NLR classes. MNLs are genes with domains or motifs specific to TNLs and CNLs. NBS domain type is labeled in the MNL section to highlight TNL-specific vs CNL-specific sequences

Lines in bold indicate sums of each category

**Fig. 1:**
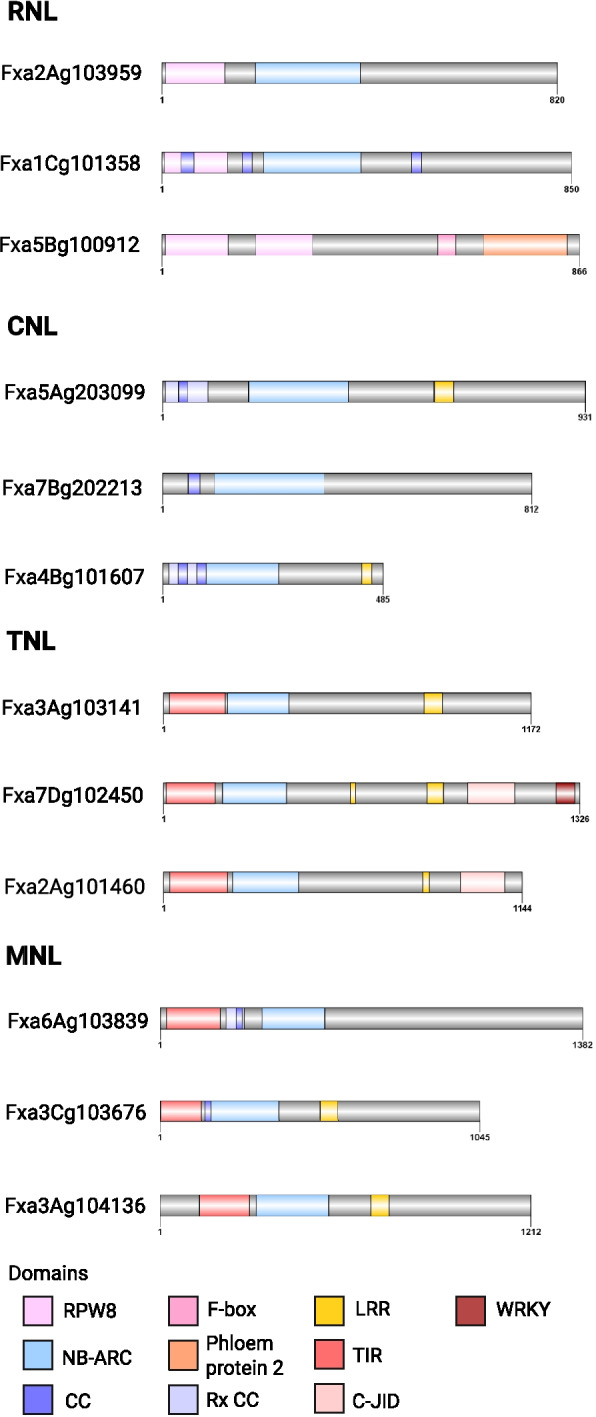
Representative functional domain architectures in different subgroups. This diagram depicts the presence and locations of canonical and non-canonical functional domains in three representative genes each for the RNL, CNL, TNL, and MNL subgroups. The length of each gene diagram is proportional to peptide length

**Fig. 2:**
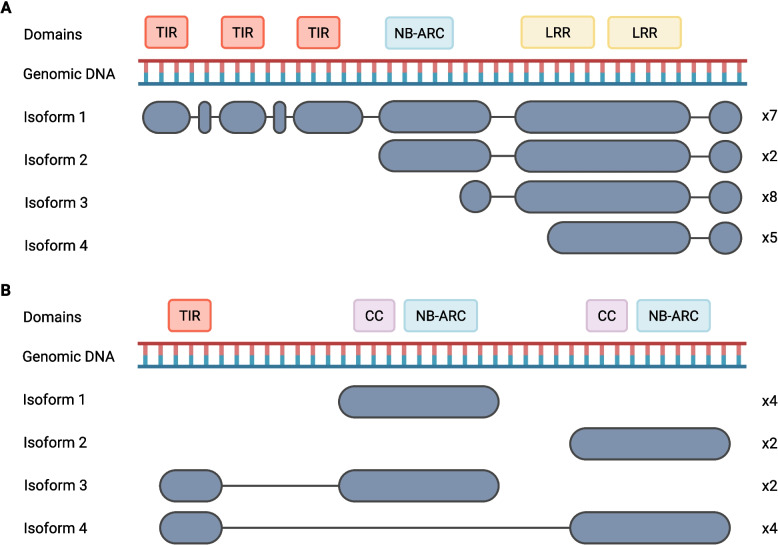
Full-length transcript data supports MNL architecture and reveals varied architecture among isoforms. These diagrams depict the isoforms of (**A**) Fxa5Ag200278 and (**B**) Fxa1Cg100403 that were observed with full length cDNA sequence data, as well as the canonical functional domains encoded by each isoform. Read number is shown to the right of each isoform. Only isoforms observed in multiple reads were included in the diagrams

Motif and functional domain analyses resulted in classification of 230 genes as CNLs, 27 of which are full-length; 298 as TNLs, 62 of which are full-length; and 99 as RNLs, none of which were predicted to have LRR domains (Table 1, Additional File 4: Table S4). There are also 106 genes predicted to lack an N-terminal signaling domain (Table 1, Additional File 4: Table S4). While all of these structures have been observed in past studies, an additional 94 genes were observed with a previously unreported architecture (Table 1, Additional File 4: Table S4). These genes were predicted to have both CNL-specific and TNL-specific sequences. Specifically, this included genes predicted to have a TIR domain in combination with a CC domain and/or a CNL-type NB-ARC domain. The CNL-type NB-ARC domains were identified based on the presence of the CNL-specific RNBS-A and RNBS-D motifs. These genes were denoted Mixed-architecture NLRs, or MNLs. Sixty of the 94 genes were predicted to be full-length. Figure 1 shows commonly observed functional domain architectures of genes from this subgroup compared to those of the three NLR classes, as determined based on motif quantification (Additional File 5: Table S5).

In order to verify that the novel architecture was not an assembly or annotation artifact, we used full-length transcript data generated with PacBio Iso-Seq technology to investigate MNL gene annotations. The Iso-Seq data confirmed existing gene models, indicating that TNL-specific sequences and CNL-specific sequences really are present in the same genes (Fig. 2). In some cases, multiple isoforms of these genes were observed, contributing to NLRs of varying sizes and complexities. In the case of Fxa1Cg100403, isoforms were present that could encode either a CNL or MNL depending on inclusion or exclusion of the TIR-encoding exon (Fig. 2B).

**Fig. 3:**
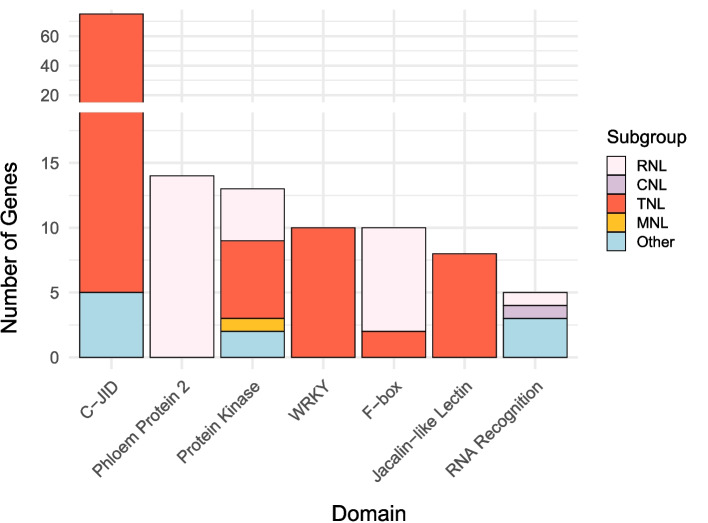
Commonly observed non-canonical domains. This histogram shows the frequency of the most commonly observed non-canonical domains, and in which subgroups they were observed. To be considered commonly observed, domains had to be predicted in five or more genes

Among all NLR classes, it is common to observe additional non-canonical domains, usually at the C-terminus. This includes any domain that is not a CC, RPW8, TIR, NB-ARC, or LRR domain, even if it commonly occurs in NLRs. In this study, 57 unique non-canonical domains were observed among 174 putative NLR genes (Additional File 3: Table S3, Additional File 5: Table S5). Most of these domains were rarely observed, with only seven present in at least five genes and only three present in more than ten genes. The most common was the C-JID domain, also known as the post-LRR domain, which was observed in 75 genes, 70 of which are TNLs (Fig. 3). The second most common was the phloem protein 2 domain, which is present in 14 genes, all of which are RNLs (Fig. 3). The third was the protein kinase domain, which is present in 12 genes of four different subgroups (Fig. 3). All of these domains have been observed in NLRs in past studies and can have different effects on NLR function [68–71].

### Phylogenetic analysis of strawberry NLRs

**Fig. 4:**
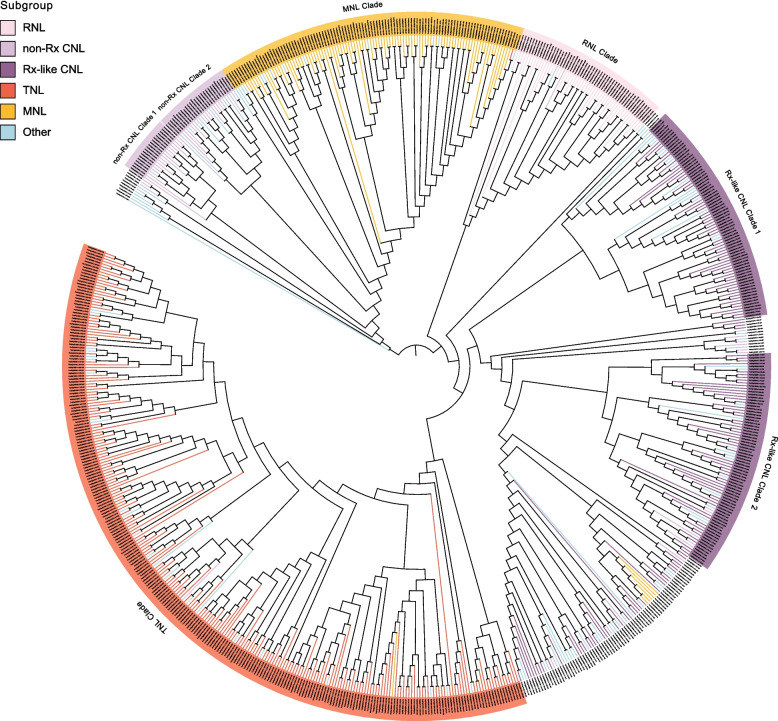
Cladogram of NB-ARC domains from strawberry NLRs. Maximum likelihood tree with branch length differences turned off. Each leaf is labeled with the gene name and the coordinates of the NB-ARC domain within the gene. Branch colors correspond to the class-associated subgroup of the gene. Annotated clades are significantly enriched for a particular subgroup

As the NB-ARC domain tends to be highly conserved according to NLR class, the sequences of these domains were used for generation of a maximum likelihood phylogenetic tree (Fig. 4). The tree is displayed as a cladogram to maximize visibility of branch color, which is indicative of the subgroup to which each gene was assigned, while the phylogram is available in the supplemental data (Additional File 6: Fig. S1). A tree with bootstrap values shown is also available in the supplemental data (Additional File 7: Fig. S2). The tree includes seven annotated clades that are statistically significantly enriched for one subgroup, based on enrichment testing with permutation (*p*-value = 9.99e-5 for each clade). It can be inferred that NBS-only genes located in one of the annotated clades likely arose from the corresponding subgroup, subsequently losing one or more domains. Analysis with the MEME Suite [34] revealed two motifs specific to the NB-ARC domains of genes in the MNL-enriched clade (Additional File 8: Table S6, Additional File 9: Fig. S3). In addition to the large, annotated MNL clade, there is a small group of MNLs that appears to be most closely related to the Rx-like CNLs, which have a particular type of CC N-terminal domain first identified in potato [72, 73]. The six MNLs in this group are all predicted to have this Rx CC domain, while none of the MNLs in the MNL-enriched clade are predicted to have it. The combined phylogenetic, domain-based, and motif-based evidence suggests multiple origins of the MNL architecture.

### Comparison of NLRs among twelve plant species

**Fig. 5:**
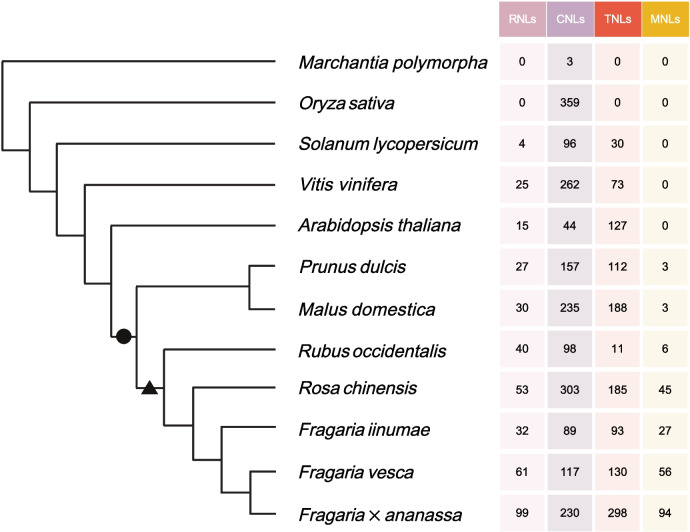
Classification of NLRs in twelve plant species. Relationships among species are shown by the cladogram on the left. The numbers of RNLs, CNLs, TNLs, and MNLs for each species are shown in the table on the right. The circle on the cladogram indicates the presence of MNL genes and the triangle indicates the presence of MNLs containing the two MNL-specific motifs identified in this study

To get a broader picture of NLR evolution leading up to the patterns observed in octoploid strawberry, we identified and classified NLRs in eleven additional species (Fig. 5, Additional File 10: Table S7). We used six species from the Rosaceae family, including *F. vesca*, *F. iinumae*, *R. chinensis* (Rose), *R. occidentalis* (Black Raspberry), *M. domestica* (Apple), and *P. dulcis* (Almond). Additionally, we included *A. thaliana* (Arabidopsis) from the Brassicaceae family, *V. vinifera* (Grape) from the Vitaceae family, *S. lycopersicum* (Tomato) from the Solanaceae family, *O. sativa* (Rice) from the Poaceae family, and *M. polymorpha* from the Marchantiaceae family. The numbers of NLRs in each subgroup varied greatly among the different species, suggesting a considerable degree of intraspecific gene loss and duplication. Despite this variation, there appears to be a clear boundary of MNL observation, which occurs between Arabidopsis and the Rosaceous species (Fig. 5). The observed boundary is unlikely to be caused by differential power of identification of MNLs, due to consistency in identification methods and genome qualities. The Arabidopsis ‘TAIR10’ genome has a high scaffold N50 of 23.5 Mb and BUSCO score of 99.3%, and the ‘FaRR1’ genome has an N50 of 23 Mb after scaffolding and gap-filling as well as a BUSCO score of 98.1% [18, 74]. However, a lack of MNLs in the non-Rosaceous species included in this study is not proof that this architecture does not exist outside of Rosaceae. Future studies of NLR evolution would be needed to improve resolution of MNL origins.

We further examined the MNLs identified in other species to determine their similarity to those in *F.* *×* *ananassa*. We searched for the two motifs specific to the MNL clade in Fig. 4 as well as for Rx CC domains, which were found only in the smaller group of MNLs. The two MNL-specific motifs were found in MNLs of species in the Rosoideae subfamily but not in *P. dulcis* or *M. domestica* (Fig. 5, Additional File 11: Table S8). Rx CC domains were detected in seven *R. chinensis* MNLs and two *F. vesca* MNLs. These two characteristics were mutually exclusive among all of the MNLs identified in this study, further supporting the hypothesis that the two groups of MNLs have separate origins.

### Gene clusters

In accordance with methods described in previous studies, NLR gene clusters are defined here as groups of genes in which every NLR is within 200 kb of another NLR [28, 49]. These groups can arise by multiple mechanisms including tandem duplications, segmental duplications, and unequal crossing over events [15, 16]. In the ‘FaRR1’ strawberry genome, 61.4% (508) of NLRs are grouped into 151 distinct clusters (Additional File 12: Table S9).

RNLs have the highest clustered to non-clustered ratio, with 74.7% (74) of RNLs existing in clusters, compared to 72.3% (68) of MNLs, 62.1% (185) of TNLs, and 52.2% (120) of CNLs. RNLs are primarily found in clusters containing only RNLs or containing both RNLs and TNLs. MNLs, the next most frequently clustered group, were found to cluster primarily with TNLs. 51.0% (77) of the clusters are heterogeneous, meaning they contain NLRs from multiple subgroups.

### Selective pressure

In order to investigate the selective pressures acting on strawberry NLRs, we calculated synonymous substitution rate (dS) and non-synonymous substitution rate (dN) for homologous NLR gene pairs, including both homoeologs and paralogs. A low proportion of non-synonymous mutations (<1) is indicative of purifying selection while a high proportion (>1) is indicative of diversifying selection [75, 76]. dN/dS was also calculated for three other gene families: the highly diverse cytochrome P450 (CYP) family, as well as aconitase (ACO) and oxoglutarate dehydrogenase (OGDH) genes, which encode highly conserved proteins that function in the TCA cycle [77–79]. These families serve as references for what the spectrum of dN/dS values looks like for gene families under diversifying and purifying selection, respectively.

**Fig. 6:**
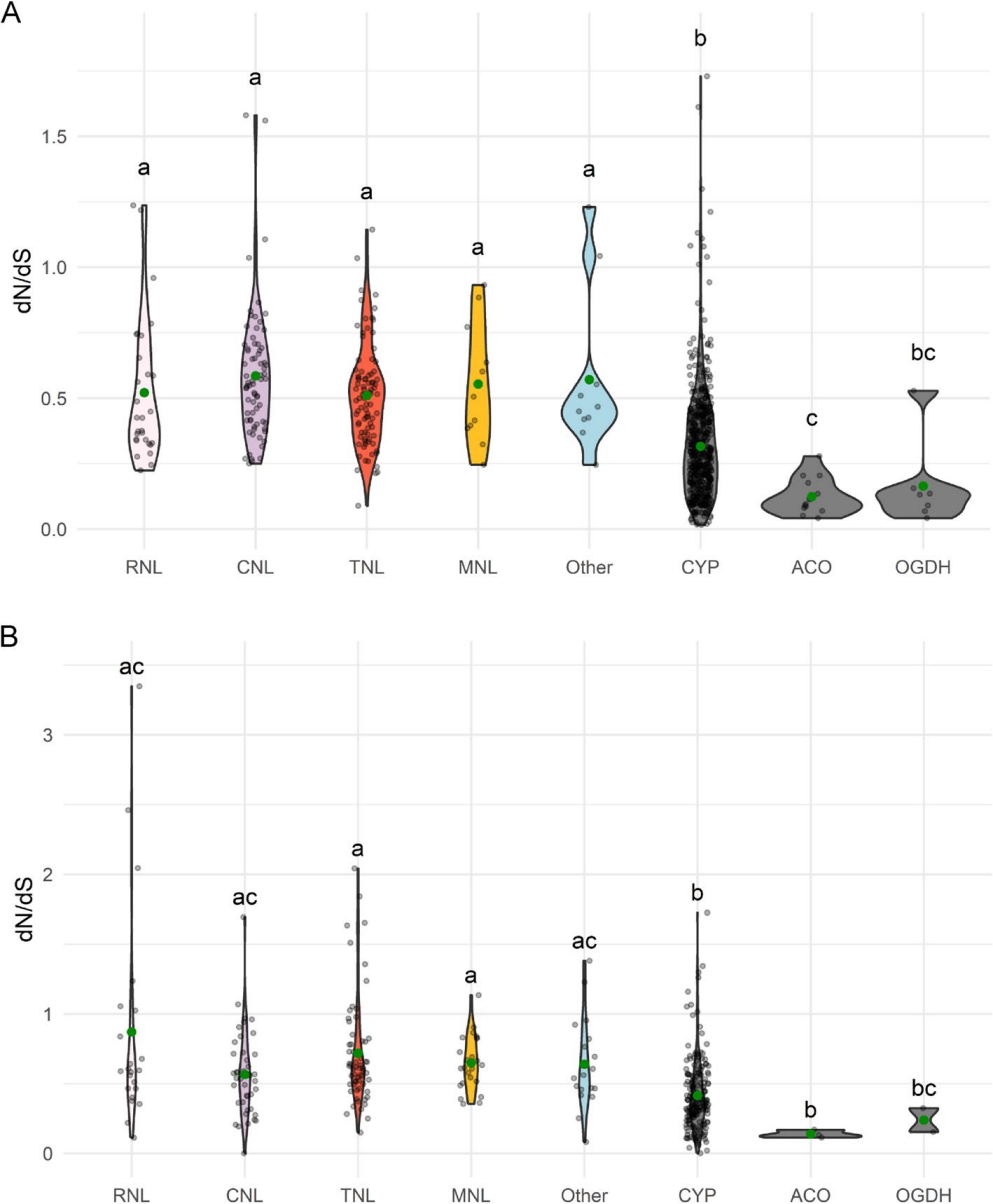
dN/dS of NLR subgroups compared to other gene families in strawberry. The violin plots show the distribution of dN/dS values for (**A**) homoeologs and (**B**) paralogs within the five NLR subgroups, the diverse cytochrome P450 (CYP) family, and the conserved aconitase (ACO) and oxoglutarate dehydrogenase (OGDH) genes. The raw data is shown as jittered points in which each point represents a comparison between two homologous genes. The green points represent the means for each group. dS values above 3 and dN/dS values above 6 were filtered as outliers. The letters denote statistically separate groups at *p<* 0.05

Homoeologous gene pairs represent duplication events that have resulted from whole genome duplication through hybridization. Assessing dN/dS of these gene pairs provides insight into how the allo-octoploid background of cultivated strawberry has impacted selective pressure on NLRs compared to other gene families. The mean dN/dS for homoeologous CYPs was 0.32, while the means for OGDH and ACO genes were 0.16 and 0.12, respectively (Fig. 6A, Additional File 13: Table S10). The mean dN/dS for the homoeologous NLRs was higher than that for all of these gene families, at 0.54 (Fig. 6A, Additional File 13: Table S10). Of the different NLR subgroups, CNLs had the highest mean dN/dS at 0.59, followed by 0.57 for NBS-only NLRs, 0.55 for MNLs, 0.52 for RNLs, and 0.51 for TNLs, each of which was significantly greater than the reference groups (Dunn’s non-parametric post-hoc test *p<* 0.05, Fig. 6A, Additional File 13: Table S10).

Analysis of paralogous gene pairs, which are identified based on sequence similarity, show even higher NLR dN/dS values, with a mean of 0.68, compared to 0.41 for CYPs, 0.14 for ACO genes, and 0.24 for OGDH genes (Fig. 6B, Additional File 13: Table S10). Of the NLR subgroups, the RNLs had the highest mean dN/dS among paralogous gene pairs at 0.87, followed by 0.72 for TNLs, 0.65 for MNLs, 0.64 for NBS-only genes, and 0.57 for CNLs (Fig. 6B, Additional File 13: Table S10). This is a nearly opposite order compared to the means of the homoeologous NLR gene pairs. However, in both sets of gene pairs, there is no statistically significant difference between the NLR subgroups, while there are significant differences between the NLRs and most or all of the other gene families (Dunn’s non-parametric post-hoc test *p<* 0.05, Fig. 6). A further breakdown of dN/dS distributions can be found in Additional File 14: Fig. S4, which displays violin plots for 0.2 dS bins, separating out recent and distant gene duplications. Most of the gene pairs have dS < 0.2, and in most cases, mean dN/dS is similar among bins (Additional File 14: Fig. S4).

### Differential gene expression analysis

**Fig. 7:**
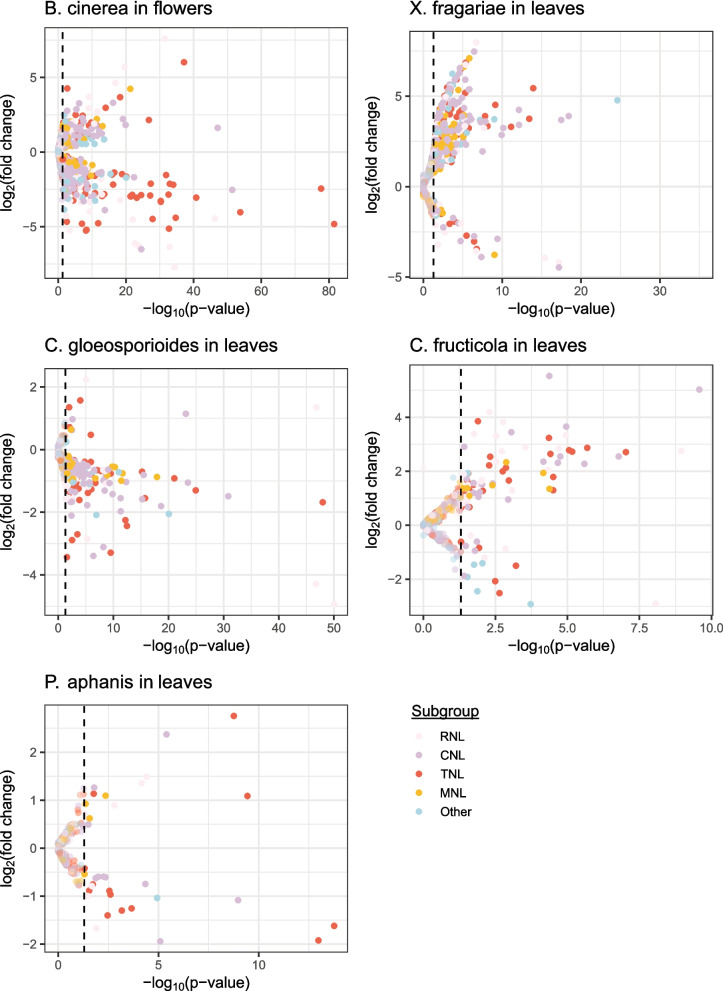
Differential NLR expression across various infection expression datasets. Scatterplots of log$_2$ fold change (L2FC) for infection treatment compared to -log$_{10}$ *p*-value of all NLR genes under infection with *B. cinerea*, *X. fragariae*, *C. gloeosporioides*, *C. fructicola*, or *P. aphanis* in various tissues. For datasets with multiple time points, the time point with the most significant *p*-value is plotted. Positive L2FC indicates higher expression under infection and negative L2FC indicates lower expression under infection when compared to water treatment. Dashed vertical line represents *p*-value significance threshold of 0.05. NLR genes (dots) are colored by subgroup. All L2FC values underwent shrinkage by “apeglm”

As a preliminary exploration of NLR function, we assessed whether strawberry NLRs are expressed during infection using published datasets of strawberry flowers inoculated with *Botrytis cinerea* [58], and strawberry leaves inoculated with *Xanthomonas fragariae* [56, 57], *Colletotrichum gloeosporioides* [55], *Colletotrichum fructicola* [54], or *Podosphaera aphanis* [59] (Additional File 15: Table S11). Of the 827 NLRs identified, 586 (70.9%) were significantly differentially expressed in at least one of five infection datasets (Additional File 16: Table S12). Per NLR subgroup, 58.5% to 81.9% of NLRs were significantly differentially expressed in at least one infection dataset, with the lowest percentage being for NLRs that lack an N-terminal signaling domain and the highest being for MNLs (Table 2, Additional File 16: Table S12). Percentages of differentially expressed genes were not significantly different between any combination of RNL, CNL, TNL, or MNL groups. However, RNLs, CNLs, and MNLs all had significantly higher percentages of differentially expressed genes than did the NBS-only genes (*p*-value = 0.002, 0.033, and 0.002, respectively). The highest percentages of NLRs were differentially expressed during *B. cinerea*, *X. fragariae*, and *C. gloeosporioides* infection. These genes were mostly induced during *X. fragariae* infection and repressed during *C. gloeosporioides* and *B. cinerea* infection (Fig. 7).

**Table 2:** Percentage of NLRs per subgroup significantly differentially expressed across infection datasets

Pathogen	RNL (%)	CNL (%)	TNL (%)	MNL (%)	Other (%)
*B. cinerea*	55.6	54.3	54.7	56.4	46.2
*X. fragariae*	38.4	42.2	31.9	53.2	31.1
*C. gloeosporioides*	34.3	30.4	20.5	34.0	16.0
*C. fructicola*	26.3	10.0	10.4	7.4	6.6
*P. aphanis*	8.1	4.4	4.4	4.3	0.9
DE in at least one dataset	74.7	74.3	67.8	81.9	58.5

## Discussion

Nucleotide-binding leucine-rich-repeat receptor proteins comprise a large, highly diverse family including three anciently diverged classes [6, 80]. These proteins play an important role in effector-triggered immunity by specifically recognizing pathogen effector molecules and mounting an immune response. In this study, the octoploid strawberry ‘FaRR1’ reference genome was used for in silico identification and analysis of NLRs, resulting in identification of 827 putative NLR genes. One hundred forty-nine of these are full-length NLRs, indicating that these genes are the most likely to be functional, although some NLRs lacking LRR or N-terminal domains may be functional as well.

Previous work has shown that the RNL, CNL, and TNL classes of NLRs diverged over 200 million years ago, with all three present in the common ancestor of angiosperms but expanding at different rates and times over evolutionary history [6]. RNLs have been the most evolutionarily conserved, leading to much lower numbers of RNL genes than CNL and TNL genes in most plants [6]. This is consistent with the findings of this study, in which only 12% of the identified NLRs belong to the RNL class, compared to 28% CNLS and 36% TNLS.

The greater expansion of the CNL and TNL classes contributes to diversity among these genes, as gene duplication events can allow mutations to accumulate with reduced pressure from purifying selection [81]. This study found that strawberry NLRs have a mean dN/dS of 0.59 for homoeologous gene pairs and 0.68 for paralogous gene pairs, both of which are significantly higher than respective means for the diverse cytochrome P450 family. This indicates that the NLR gene family in strawberry has undergone very low levels of purifying selection, especially for small-scale duplications. In total, 43 NLR gene pairs had dN/dS values over one, demonstrating that many strawberry NLRs are experiencing diversifying selection.

Moreover, tandem and segmental duplications can contribute to NLR gene clusters, which can be further drivers of NLR diversity. In this study, 61.4% of the NLRs identified were grouped into 151 clusters, roughly half of which contained multiple NLR subgroups. This is a similar but slightly lower proportion compared to Arabidopsis and potato, each of which has about 73% of NLR genes in clusters [14, 28]. Clustering of NLRs in the genome both promotes physical associations between NLR proteins and contributes to diversity in NLR sequences, such as through polymerase slippage in repetitive regions [7, 12, 28]. Sequence exchange in gene clusters has been shown to lead to the addition of non-canonical, or integrated, domains, usually at the C-terminus of NLRs [7, 12]. We observed 57 unique non-canonical domains among 175 NLR genes, the most frequent of which was the C-JID domain. This domain is known to be common in TNLs, and it has been shown to play a role in pathogen recognition and NLR complex formation for some of these proteins [68, 69]. Other integrated domains, such as protein kinase and WRKY domains, can function as baits to increase effector binding or as guards that bind PTI-related genes in order to recognize their interactions with effectors [12, 71, 82].

Among the diverse NLR architectures identified, one configuration stood out as particularly unusual. We discovered a previously unreported hybrid architecture combining both CNL-specific and TNL-specific sequences. Of the 94 genes with this architecture, each includes a TIR domain together with a CC domain and/or a CNL-type NB-ARC domain. Twenty-seven of these include a CC domain while only one harbors a TNL-type NB-ARC domain. The coexistence of CNL- and TNL-specific sequences in the same genes and isoforms was confirmed by full-length transcript evidence from PacBio Iso-Seq data. In several cases, the Iso-Seq reads revealed complex alternative splicing, including at least one example in which MNL- and CNL-type isoforms were produced from the same locus depending on inclusion of the TIR-encoding exon (Fig. 2). This suggests that domain exchange and splicing may jointly contribute to the diversification of NLR structure and function in strawberry.

Phylogenetic analysis separated the MNLs into two main groups: a large clade labeled in Fig. 4 as “MNL Clade”, as well as a smaller clade of six genes grouped among Rx-like CNLs. The MNLs in these groups have different distinguishing features including two motifs specific to the NB-ARC domains of MNLs in the larger clade and Rx-type CC domains only in the MNLs of the smaller clade. These features indicate that the two groups likely have different origins. These may have been two different sequence integration or exchange events in which TIR domains were integrated into the N-terminal regions of CNL genes during DNA replication. The syntenic relationships of genes in the smaller group indicates that duplications were primarily a result of whole-genome duplication through hybridization, while the larger group has undergone a greater number of duplications, evolving its own unique motifs and sometimes losing CC domains.

Analysis of NLRs in other plant species revealed that MNLs originated before the evolution of strawberry, with MNLs present in every species investigated in the Rosaceae family and the MNL-specific motifs present in every species investigated in the Rosoideae subfamily (Fig. 5). No MNLs were identified in the five non-Rosaceous species examined. Among all of the MNLs in the included species, presence of the MNL-specific motifs and presence of the Rx CC domain remained mutually exclusive, further supporting the hypothesis of separate origins of these groups.

While the effect of the mixed architecture on NLR function is unknown, analysis of gene expression suggests that the genes probably have not lost function entirely. Not only are the genes expressed, but this subgroup has the highest percentage of genes that are differentially expressed in response to at least one of the pathogens included in this study at 81.9% compared to 74.7% for RNLs, 74.3% for CNLs, 67.8% for TNLs, and 58.5% for other NLRs (Table 2). When accounting for the different percentages for all pathogen studies included, the MNLs have significantly greater proportions of differential expression compared to the other NLRs, which lack N-terminal domains and are less likely to be functional. Although this does not prove a direct role in pathogen resistance, it does suggest a role in the general strawberry immune response. However, further experimentation is still needed in order to validate the novel architecture and investigate how it arose and how it affects NLR function.

## Conclusion

We conducted a comprehensive genome-wide analysis of NLR genes in *F.* *×* *ananassa*. By integrating motif- and domain-based annotation with phylogenetic analysis, we identified 827 putative NLRs and discovered a previously undescribed hybrid architecture that integrates features of both CNL and TNL classes. This arrangement appears to have evolved multiple times in the Rosaceae family, suggesting ongoing diversification and structural innovation in this lineage. The widespread transcriptional responsiveness of these genes, particularly within the MNL subgroup, underscores their potential importance in strawberry immunity. Collectively, our results highlight an unexpected level of structural and functional diversity among strawberry NLRs and provide a genomic framework for future studies aimed at exploring the molecular basis of disease resistance in Rosaceae.

## Supplementary information

Additional file 1. Table S1 NLR-Annotator Raw Output F ananassa. Table of NLR loci and motifs returned by NLR-Annotator.

Additional file 2. Table S2 Blast Results. Table of tblastn results with the results of each search on a different, labeled sheet. The last sheet compiles all results not matching a previously identified NLR and includes notes regarding whether to add them to the gene list.

Additional file 3. Table S3 Interpro Raw Output. Table of predicted functional domains returned by Interpro. Each subgroup of genes is on a separate, labeled sheet.

Additional file 4. Table S4 NLR Genes Bed. NLR gene names and coordinates in bed file format. Each subgroup of genes is on a separate, labeled sheet. Additionally, the fifth column of the CNL sheet includes metadata on CC type.

Additional file 5. Table S5 Quantified Domains. The number and percentage of genes with each domain identified in each subgroup.

Additional file 6. Fig. S1 NBS Phylogram. Phylogenetic tree from Fig. 4 displayed as a phylogram, in which branch length corresponds to sequence similarity. Branch color and clade annotations are the same as in Fig. 4.

Additional file 7. Fig. S2 NBS Cladogram with Bootstraps. Phylogenetic tree from Fig. 4 with bootstrap numbers displayed on the tree.

Additional file 8. Table S6 MEME Raw Output. Raw output from MEME motif search including motif sequences identified, lists of genes containing each motif with *p*-values, and lists of motifs in each gene.

Additional file 9. Fig. S3 MNL-Specific Motifs. (A) Phylogenetic tree from Fig. 4 with genes containing the two MNL-specific motifs indicated with green circles next to the labels, (B) logo diagrams of the two MNL-specific motif sequences, and (C) a section of a multiple sequence alignment of NB-ARC domains from three genes each of the CNL, TNL, and MNL subgroups, with positions of MNL-specific motifs indicated with yellow circles.

Additional file 10. Table S7 NLR-Annotator Raw Output Other Species. Tables of NLR loci and motifs returned by NLR-Annotator for each of the eleven additional species in the species tree. Output for each species is on a separate, labeled sheet.

Additional file 11. Table S8 MAST Raw Output. Raw output from MAST motif search including lists of genes containing the provided motif sequences with *p*-values and lists of motifs in each gene.

Additional file 12. Table S9 Gene Clusters. Table including cluster names and coordinates, gene names, and gene subgroups for each clustered gene.

Additional file 13. Table S10 dNdS. Table of synonymous and nonsynonymous mutation rates for syntenic or paralogous gene pairs. The first two columns contain the gene names, the third column contains dN values, the fourth column contains dS values, the fifth column contains dN/dS values, and the sixth column contains NLR subgroup or gene family information.

Additional file 14. Fig. S4 dNdS of NLR subgroups and other gene families binned by dS. Violin plots of dN/dS for the NLR subgroups, cytochrome p450s, aconitases, and oxoglutarate dehydrogenases, with means shown as green points. The plots are separated based on 0.2 dS bins to show selection on older and more recent duplications. Panel A displays dN/dS for syntenic gene pairs while panel B displays dN/dS for paralogous gene pairs.

Additional file 15. Table S11 Expression Datasets. Table with additional information about expression datasets used in this study, including NCBI project numbers.

Additional file 16. Table S12 Differential Expression. Table of NLR genes with differential expression data for infection with each of five pathogens. This includes adjusted *p*-values and log2fold change in expression.

## Data Availability

The ‘FaRR1’ F. × ananassa (https://phytozome-next.jgi.doe.gov/info/FxananassaRoyalRoyce_v1_0), F. vesca (https://phytozome-next.jgi.doe.gov/info/Fvesca_v4_0_a2), A. thaliana (https://phytozome-next.jgi.doe.gov/info/Athaliana_TAIR10), V. vinifera (https://phytozome-next.jgi.doe.gov/info/Vvinifera_v2_1), S. lycopersicum (https://phytozome-next.jgi.doe.gov/info/Slycopersicum_ITAG4_0), O. sativa (https://phytozome-next.jgi.doe.gov/info/Osativa_v7_0), and M. polymorpha (https://phytozome-next.jgi.doe.gov/info/Mpolymorpha_v3_1) genomes are available through Phytozome. The F. iinumae (https://www.rosaceae.org/species/fragaria_iinumae/genome_v1.0), R. chinensis (https://www.rosaceae.org/analysis/282), R. occidentalis (https://www.rosaceae.org/analysis/268), M.domestica (https://www.rosaceae.org/species/malus/malus_x_domestica/genome_GDDH13_v1.1), and P. dulcis (https://www.rosaceae.org/analysis/295) genomes are available through the Genome Database for Rosaceae. The RNAseq and Iso-Seq data are accessible through the NCBI SRA (https://www.ncbi.nlm.nih.gov/sra). Custom scripts used for this work are available on GitHub (https://github.com/aliciasillers/NLR-Identification).
