# Supplementary material for: Genome-wide analysis of the NLR gene family in strawberry reveals a novel immune receptor architecture in Rosaceae
Source: BMC Genomics. 2026 Jun 5;27:666. doi: 10.1186/s12864-026-13005-1 (PMC13465223; doi:10.1186/s12864-026-13005-1)
Supplement: Supplementary file 1 [file 12864_2026_13005_MOESM14_ESM.pdf]

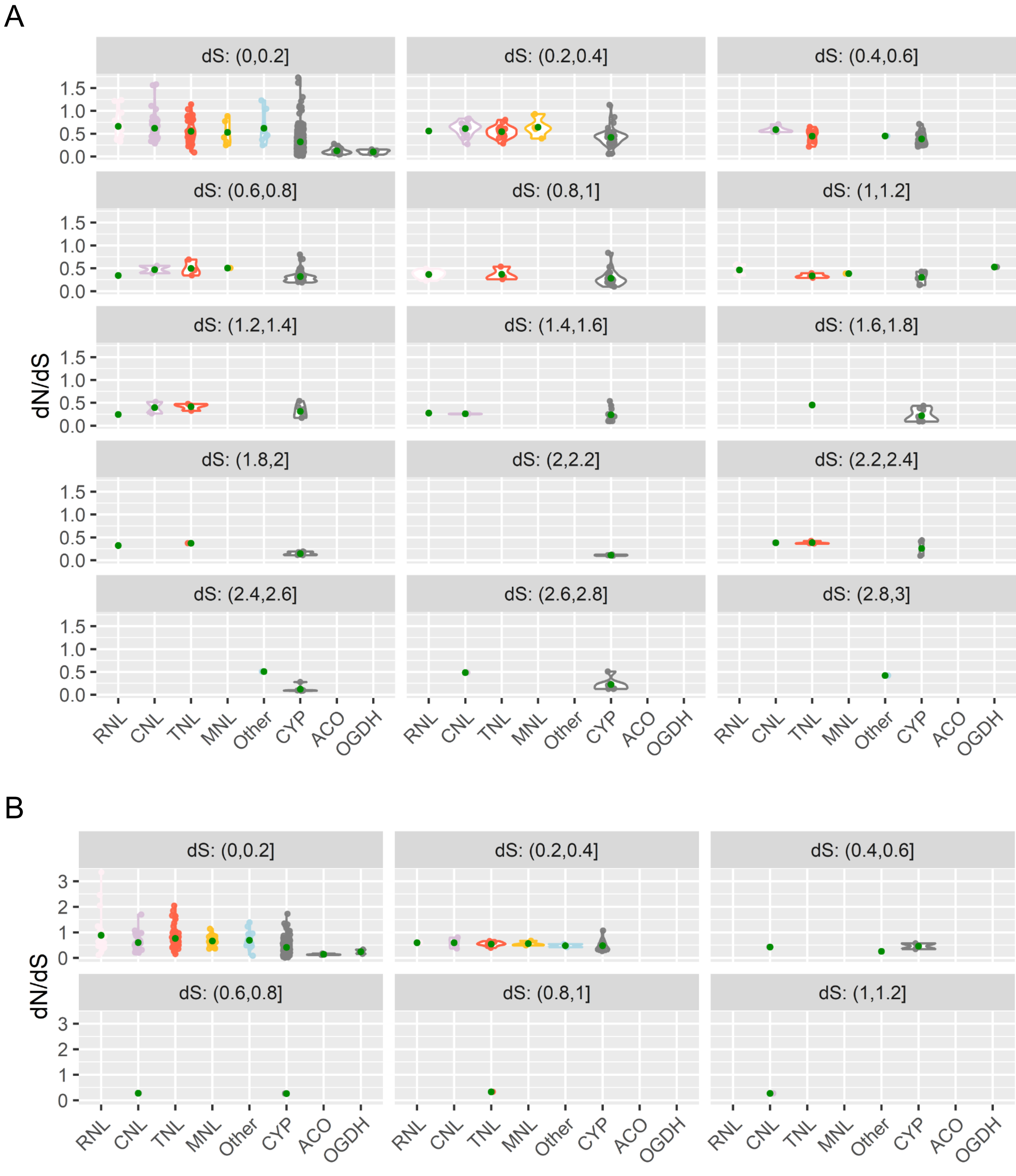
A
dS: (0,0.2]
dS: (0.2,0.4]
dS: (0.4,0.6]
dS: (0.6,0.8]
dS: (0.8,1]
dS: (1,1.2]
dS: (1.2,1.4]
dS: (1.4,1.6]
dS: (1.6,1.8]
dS: (1.8,2]
dS: (2,2.2]
dS: (2.2,2.4]
dS: (2.4,2.6]
dS: (2.6,2.8]
dS: (2.8,3]
dN/dS
0.0
0.5
1.0
1.5
RNL
CNL
TNL
MNL
Other
CYP
ACO
OGDH
B
dS: (0,0.2]
dS: (0.2,0.4]
dS: (0.4,0.6]
dS: (0.6,0.8]
dS: (0.8,1]
dS: (1,1.2]
dN/dS
0
1
2
3
