# Supplementary material for: Genome-wide analysis of the NLR gene family in strawberry reveals a novel immune receptor architecture in Rosaceae
Source: BMC Genomics. 2026 Jun 5;27:666. doi: 10.1186/s12864-026-13005-1 (PMC13465223; doi:10.1186/s12864-026-13005-1)
Supplement: Supplementary file 2 [file 12864_2026_13005_MOESM6_ESM.pdf]

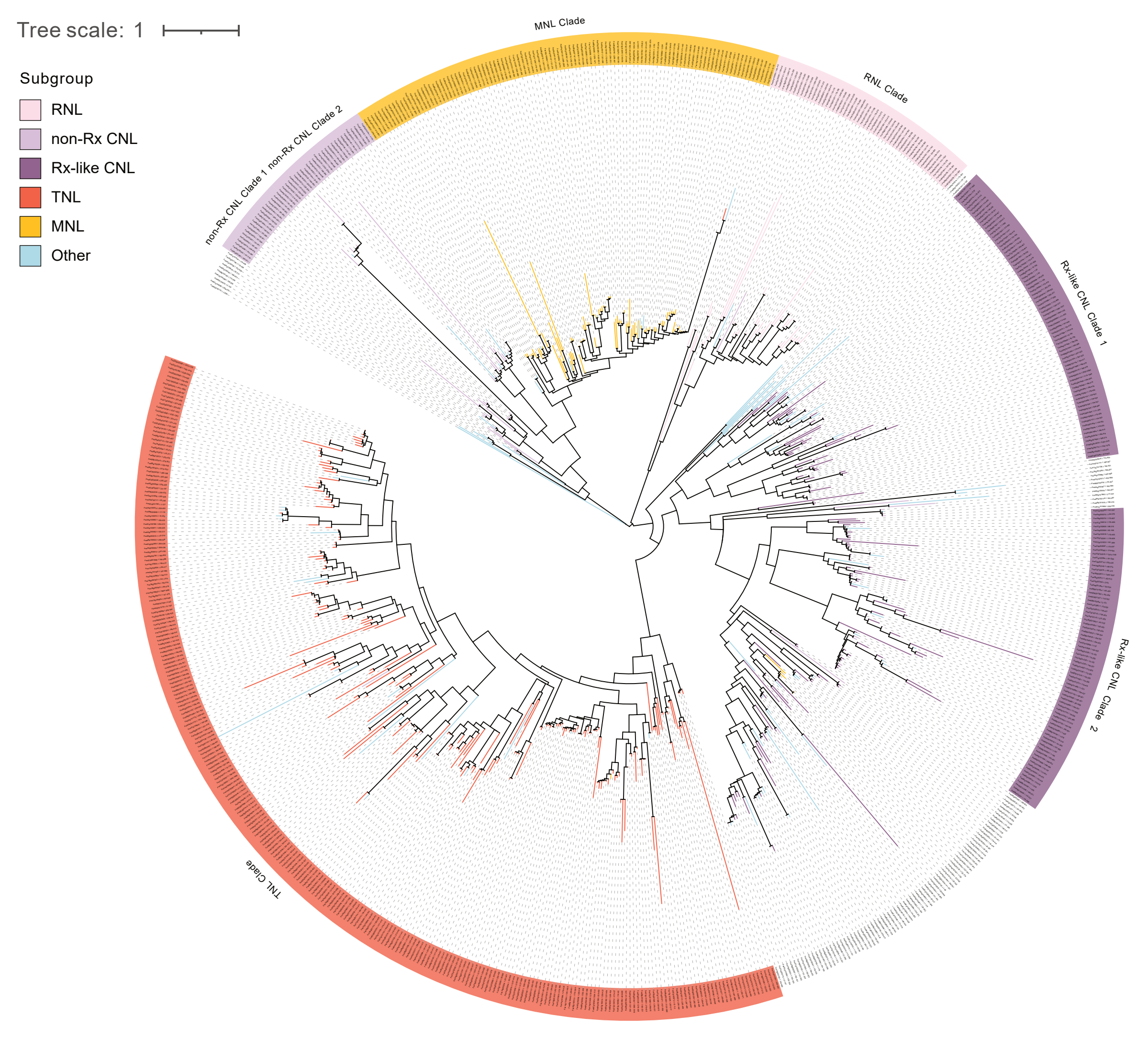
Tree scale: 1
Subgroup
RNL
non-Rx CNL
Rx-like CNL
TNL
MNL
Other
MNL Clade
RNL Clade
Rx-like CNL Clade 1
Rx-like CNL Clade 2
TNL Clade
non-Rx CNL Clade 1
non-Rx CNL Clade 2
