# Supplementary material for: Genome-wide analysis of the NLR gene family in strawberry reveals a novel immune receptor architecture in Rosaceae
Source: BMC Genomics. 2026 Jun 5;27:666. doi: 10.1186/s12864-026-13005-1 (PMC13465223; doi:10.1186/s12864-026-13005-1)
Supplement: Supplementary file 4 [file 12864_2026_13005_MOESM9_ESM.pdf]

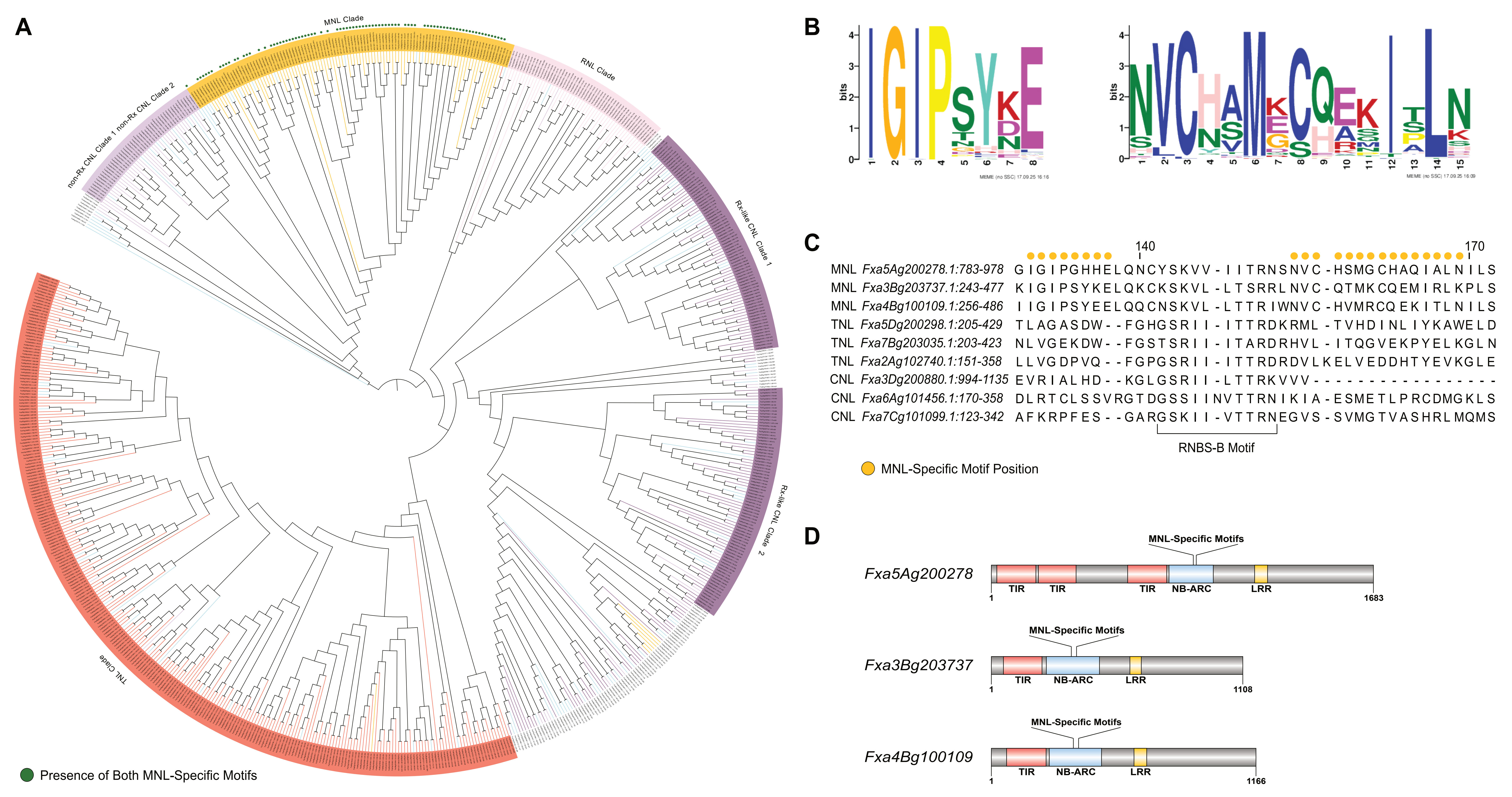
A
MNL Clade
RNL Clade
non-Rx CNL Clade 1
non-Rx CNL Clade 2
Rx-like CNL Clade 1
Rx-like CNL Clade 2
TNL Clade
Presence of Both MNL-Specific Motifs
B
bits
MEME (no SSC) 17.09.25 16:16
MEME (no SSC) 17.09.25 16:09
C
140
170
MNL Fxa5Ag200278.1:783-978 GIGIPGHHELQNCYSKVV-IITRNSNVC-HSMGCHAQIALNILS
MNL Fxa3Bg203737.1:243-477 KIGIPSYKELQKCKSKVL-LTSRRLNVC-QTMKCQEMIRLKPLS
MNL Fxa4Bg100109.1:256-486 IIGIPSYEELQQCNSKVL-LTTRIWNVC-HVMRCQEKITLNILS
TNL Fxa5Dg200298.1:205-429 TLAGASDW--FGHGSRII-ITTRDKRML-TVHDINLIYKAWELD
TNL Fxa7Bg203035.1:203-423 NLVGEKDW--FGSTSRII-ITARDRHVL-ITQGVEKPYELKGLN
TNL Fxa2Ag102740.1:151-358 LLVGDPVQ--FGPGSRII-ITTRDRDVLKELVEDDHTYEVKGLE
CNL Fxa3Dg200880.1:994-1135 EVRIALHD--KGLGSRII-LTTRKVVV----------------
CNL Fxa6Ag101456.1:170-358 DLRTCLSSVRGTDGSSIINVTTRNIKIA-ESMETLPRCDMGKLS
CNL Fxa7Cg101099.1:123-342 AFKRPFES--GARGSKII-VTTRNEGVS-SVMGTVASHRLMQMS
RNBS-B Motif
MNL-Specific Motif Position
D
MNL-Specific Motifs
Fxa5Ag200278
TIR
TIR
TIR
NB-ARC
LRR
1
1683
MNL-Specific Motifs
Fxa3Bg203737
TIR
NB-ARC
LRR
1
1108
MNL-Specific Motifs
Fxa4Bg100109
TIR
NB-ARC
LRR
1
1166
